# Prebiotics Regulation of Intestinal Microbiota Attenuates Cognitive Dysfunction Induced by Surgery Stimulation in APP/PS1 Mice

**DOI:** 10.14336/AD.2020.0106

**Published:** 2020-10-01

**Authors:** Dengyang Han, Zhengqian Li, Taotao Liu, Ning Yang, Yue Li, Jindan He, Min Qian, Zhongshen Kuang, Wen Zhang, Cheng Ni, Xiangyang Guo

**Affiliations:** ^1^Department of Anesthesiology, Peking University Third Hospital, Beijing, China; ^2^National Institute on Drug Dependence and Beijing Key Laboratory of Drug Dependence, Peking University, Beijing, China

**Keywords:** intestinal microbiota, xylooligosaccharides, postoperative cognitive dysfunction, tight junction, inflammation

## Abstract

Emerging evidence indicates that the intestinal microbiota could interact with the central nervous system and modulate multiple pathophysiological changes, including the integrity of intestinal barrier and blood-brain barrier, as well as neuroinflammatory response. In the present study, we investigated the potential role of intestinal microbiota in the pathophysiological process of postoperative cognitive dysfunction. Six-month-old APP/PS1 mice were subjected to partial hepatectomy to establish surgery model and exhibited cognitive dysfunction. The expressions of inflammatory mediators increased and tight junction proteins (ZO-1 and Occludin) levels decreased in the intestine and hippocampus. The 16S ribosomal RNA gene sequencing showed altered β diversity and intestinal microbiota richness after surgery, including genus *Rodentibacter*, *Bacteroides*, *Ruminococcaceae_UCG_014* and *Faecalibaculum*, as well as family *Eggerthellaceae* and *Muribaculaceae*. Furthermore, prebiotics (Xylooligosaccharides, XOS) intervention effectively attenuated surgery-induced cognitive dysfunction and intestinal microbiota alteration, reduced inflammatory responses, and improved the integrity of tight junction barrier in the intestine and hippocampus. In summary, the present study indicates that intestinal microbiota alteration, the related intestinal barrier and blood-brain barrier damage, and inflammatory responses participate the pathophysiological process of postoperative cognitive dysfunction. Prebiotics intervention could be a potential preventative approach.

The intestinal microbiota profoundly affects human health through modulating the host immune and nervous systems. Altered intestinal microbiota not only impairs intestinal barrier integrity and triggers peripheral inflammatory reaction, but also induces neuroinflammatory response or disruption of blood-brain barrier (BBB) through gut-brain axis [1-3]. In addition, the increased permeability of the intestinal barrier and BBB induced by intestinal dysbiosis had been considered as pivotal causes of neurodegenerative diseases [4].

Postoperative cognitive dysfunction (POCD) is a complication of central nervous system (CNS). The typical symptoms include memory loss and orientation disorder, accompanied by decreased social activity [5]. Population with fragile brains are more likely to develop POCD, particularly in patients with Alzheimer’s Disease (AD) [6]. Although the mechanisms of POCD remain to be elucidated, neuroinflammatory response and BBB damage have been recognized as the primary pathogenic factors [7]. Ours and related studies found that peripheral inflammatory mediators induced by anesthesia and surgery could infiltrate the CNS through disrupted BBB and lead to cognitive dysfunction [8, 9]. In addition, microglia can be overactivated during CNS injury, and release cytotoxic factors and mediators, which cause subsequent damages to neurons [10]. Thus, we speculate that dysregulation of intestinal microbiota could affect systemic inflammatory response, disrupt the barrier integrity in intestine and CNS, which ultimately lead to the development of POCD.

Known as non-digestible or low-digestibility food ingredients, prebiotics could regulate intestinal microbiota and improve host health [11]. Xylooligosaccharides (XOS), a prebiotics fiber composed of 2-7 xylose molecules bound by β-1,4 glycoside bonds, has been proved to be beneficial in both clinical and animal studies, including downregulating the proportion of pathogenic bacteria of intestinal tract, decreasing peripheral and central inflammatory mediators [3, 12, 13]. The APP/PS1 mice exhibit fragile brain associated with β-amyloid elevation and behavioral abnormalities [14]. In the present study, the adult APP/PS1 mice were used to mimic the fragile brain model and partial hepatectomy under sevoflurane anesthesia was employed to induce POCD. The 16S ribosomal RNA (rRNA) gene sequencing was used to assess the concrete effects of XOS on the intestinal microbiota. In addition, the inflammation markers and integrity of barriers in both peripheral and central nervous system were investigated to explore the potential effects of prebiotics on POCD.

## MATERIALS AND METHODS

### Experimental animals and XOS supplementation

Six-month-old male APP/PS1 mice, weighing 20-25g, were used in this study. Before study, the mice were maintained on a standard housing condition (12-hour light/12-hour dark cycle, 22±2 ?, food and water ad libitum) for one week. Animal experiments were conducted in accordance with the guidelines for the laboratory animals and the protocol was approved by Peking University Biomedical Ethics Committee Experimental Animal Ethics Branch (No. LA20150041).

The mice were randomly assigned into five groups: Control (Con) group, anesthesia group, surgery (S) group, XOS group, and XOS+surgery (XOS+S) group. The mice in anesthesia group underwent 0.5 h sevoflurane anesthesia. The mice in S and XOS+S group underwent partial hepatectomy plus 0.5 h sevoflurane anesthesia. Animals in XOS and XOS+S group were fed with XOS by gavage for five weeks before and one week after surgery (150 μl/day, 10% XOS in PBS, at 9 am). The mice in Con, anesthesia and S groups were fed with PBS by gavage. Based on a previous study, 10% XOS could modulate intestinal microbiota and reduce neuroinflammatory response in obese-insulin resistant rats [3]. The duration of XOS intervention was based on several studies. In a clinical study, four weeks of prebiotics intervention could affect intestinal microbiota and improve cognition function [15]. In an animal study of LPS induced cognitive dysfunction, three weeks of prebiotics intervention could significantly ameliorate the neuroinflammatory response and improve the cognitive function [16]. Six weeks of XOS intervention in our preliminary experiment showed protective effects on postoperative cognitive dysfunction and related mechanisms.

### Anesthesia and surgery

Mice in the anesthesia group received 2.5% (1.0 MAC) sevoflurane anesthesia in fresh air/O_2_ in an anesthetizing chamber. The mice breathed spontaneously, and the sevoflurane concentration was measured continuously (Datex, Tewksbury, MA). This anesthesia protocol has been proved not significantly altered blood gas and blood pressure in a previous study [17]. Finally, the mice recovered on the thermal insulation blanket in a chamber filling with 100% oxygen until they woke up.

The mice in S and XOS+S group underwent partial hepatectomy under 2.5% sevoflurane anesthesia. The liver was exposed through a 2 cm upper midline abdominal incision. The left lateral lobe of the liver was resected. The incision was then closed with 5-0 suture and sterilized with iodophor three times followed by 0.25% bupivacaine infiltration. After surgery, the mice recovered on the thermal insulation blanket in a chamber containing 100% oxygen. The lidocaine hydrochloride gel was applied to the incision every 8 h until 2 days after surgery. Mice in Con group received no treatment. This surgery protocol has been shown not to alter liver function in a previous study [18]. The microbiota analysis, behavioral tests, liver function and glucose levels were measured two days after surgery. There were no significant differences in liver function and glucose levels among four groups.[Table T1-ad-11-5-1029]

**Table 1 T1-ad-11-5-1029:** Liver function and glucose levels after experimental interventions.

	ALT (U/L)	AST (U/L)	ALP (U/L)	TP (g/L)	ALB (g/L)	GLU (mmol/L)
Con	36.9±5.9	50.7±3.0	186.0±14.5	39.7±0.5	23.93±0.8	9.6±0.6
S	42.8±3.3	55.9±5.1	189.7±8.9	34.5±3.7	24.2±1.1	10.7±0.9
XOS+S	39.3±3.5	49.1±4.7	192.8±10.9	36.7±1.9	22.1±1.4	10.9±0.7
XOS	35.5±3.6	43.2±3.4	210.7±13.9	37.7±3.9	24.4±0.8	9.7±1.3

ALT, alanine transaminase; AST, aspartate aminotransferase; ALP, alkaline phosphatase; TP, total protein; ALB, albumin; GLU, glucose. Mean ± SEM (n =3). There were no significant differences among groups

### Morris water maze test

Spatial memory was evaluated using Morris water maze test as described previously with minor modification [18]. The water pool was divided into four sectors equally. The mouse was faced to the tank wall and placed into the water in turn from sector one to sector four with an interval rest of 10 minutes. In all trials, mice were allowed to swim until they climbed on the hidden platform. If a mouse failed to find the platform within 90 s, it was guided toward the platform and stayed there for 30 s. Mice underwent test daily for five days before and seven days after surgery. On the 3rd and 7th day after surgery, the probe trial test was performed (After the platform was removed, the mouse was allowed swimming for 90 s). The times to cross the platform region was recorded and represented as an index of memory function. The temperature of the water was maintained at 20-22 ?, and all tests were completed between 1 pm and 4 pm.

### Fecal sample DNA extraction, PCR amplification and sequencing

Two days after surgery, fresh fecal pellets were collected directly from the anal orifices, frozen immediately in liquid nitrogen and stored at -80 °C for analyses. The following experiments were conducted by Majorbio (Shanghai, China). Microbial DNA was extracted from fecal samples using the E.Z.N.A.® Soil DNA Kit (Omega Bio-Tek, Norcross, GA, USA) according to the manufacturer’s protocols. The concentration and purification of extracted DNA were determined by a NanoDrop 2000 UV-vis spectrophotometer (Thermo Scientific, Wilmington, NC, USA), the DNA concentrations were more than 50 ng/μl and the 260nm/280nm absorbance ratio of each sample were between 1.8-2.0. The quality of DNA was checked by 2% agarose gel electrophoresis. The V3-V4 hypervariable regions of the bacterial 16S rRNA gene were amplified with primers 338?F (5′-ACTCCTACGGGAGGCA GCAG-3′) and 806?R (5′-GGACTACHVGGGTWTCTA AT-3′) by GeneAmp 9700 thermocycler PCR system (Thermo Scientific, Wilmington, NC, USA). PCR reactions were conducted using the following program: 3?min of denaturation at 95?°C, 27 cycles of 30?s at 95?°C, 30?s for annealing at 55?°C, and 45?s for elongation at 72?°C, and a final extension at 72?°C for 10?min. PCR reactions were performed in triplicate in a 20?μl mixture containing 4?μl of 5?×?FastPfu Buffer, 2?μl of 2.5?mmol/L dNTPs, 0.8?μl of each primer (5?μmol/L), 0.4?μl of FastPfu Polymerase and 10?ng of template DNA. The resulting PCR products were extracted from a 2% agarose gel and further purified using the AxyPrep DNA Gel Extraction Kit (Axygen Biosciences, Union City, CA, USA) and quantified using QuantiFluor™-ST (Promega, Madison, WI, USA) according to the manufacturer’s protocol. Barcoded amplicons were pooled for library construction using the TruSeq DNA Sample Prep kit (Illumina, San Diego, CA, USA) and purified amplicons were pooled in equimolar and paired-end sequenced (2?×?300) on an Illumina MiSeq platform (Illumina, San Diego, CA, USA).

### Processing of sequencing data

The following steps were conducted by Majorbio (Shanghai, China). Raw fastq files were demultiplexed, quality filtered by Trimmomatic and merged by FLASH based on the following criteria: (i) The reads were truncated at any site that received an average quality score?<?20 over a 50?bp sliding window; (ii) The primers were exactly matched, allowing a 2-nucleotide mismatch, and reads containing ambiguous bases were removed; (iii) Sequences with overlaps of longer than 10?bp were merged according to their overlap sequence. Operational taxonomic units were clustered with a 97% similarity cutoff using UPARSE (version 7.1, http://drive5.com/uparse), and chimeric sequences were identified and removed using UCHIME. The taxonomy of each 16S rRNA gene sequence was analyzed by the RDP Classifier algorithm (http://rdp.cme.msu.edu/) against the Silva 16S rRNA database (version Silva 132/16s bacteria) with a confidence threshold of 70%. We obtained a median of 42,871 sequences per sample with a range of 31,802-50,692 and used 20,000 sequences per sample for rarefaction.

### Bioinformatics analysis

The β diversity analysis was used to distinguish the similarities or differences among different samples in this study. QIIME was used for PCoA analysis, obtaining a distance matrix using weighted UniFrac algorithm. R language tools were used to create PCoA curve. The relative abundances of taxa were generated for each sample using QIIME, and the inferred absolute abundances of taxa were calculated by multiplying the 16S rRNA copy number and the taxonomic relative abundance within a given sample.

### Lysis and protein quantification

Two days after surgery, mice were anesthetized and perfused transcranially with ice-cold saline. The intestinal and brain tissues were removed, and the hippocampus was dissected. The harvested tissues were extracted on ice using RIPA lysis buffer plus protease inhibitors, as described in our previous studies [19]. The lysates were collected, centrifuged at 4 ? for 15 min at 16,000 g. The protein levels in the supernatant were measured by a BCA assay kit (Pierce, Rockford, IL, USA).

### Western blot analysis

The supernatant was collected followed by western blot analysis. The proteins (30-50 µg) were transferred into polyvinylidene fluoride microporous membranes. Then, the membranes were blocked with 5% non-fat milk in TBST for 2 h at room temperature and incubated overnight on lab shaker at 4 ? with the following primary antibodies: rabbit monoclonal to Iba-1 (1:1000 dilution, Abcam, Cambridge, UK), goat polyclonal to TREM2 (1:1000 dilution, Abcam, Cambridge, UK), rabbit polyclonal to ZO-1 (1:1000 dilution, Thermo Scientific, Wilmington, NC, USA), rabbit monoclonal to Occludin (1:1000 dilution, Abcam, Cambridge, UK), mouse monoclonal to Claudin-5 (1:500 dilution, Thermo Fisher Scientific, Wilmington, NC, USA), or mouse monoclonal to GAPDH (1:5000 dilution, Abcam, Cambridge, UK). The membranes were incubated with horseradish peroxidase-coupled secondary antibody (1:500 dilution, Abcam, Cambridge, UK). The immunoreactivity was detected using enhanced chemiluminescence and visualized using Image Lab software (Bio-Rad Laboratories, Hercules, CA, USA). The intensity of the protein bands was quantized by Image J software and the relative expression levels of protein were normalized by the ratio of the target protein to GAPDH.

### ELISA assay

The collected supernatants were analyzed for IL-1β, IL-6, TNF-α and IL-10 using Elisa kit (NeoBioscience, Beijing, China) according to the instructions provided by the manufacturer. The absorbance read at 450 nm using a micro-plate reader and then calculated as a concentration using a standard curve. The inflammatory mediators in the tissue were expressed as the ratio of inflammatory mediator concentration to total protein concentration.

### Immunofluorescence

Immunofluorescence was used to determine the expression levels of endogenous Iba-1 protein in CA1 region and dentate gyrus (DG) of hippocampus. CA1 region contains the pyramidal cells located closest to the subiculum of hippocampus, DG is part of the hippocampal formation in the temporal lobe, and these regions all participate in learning and memory processes [20, 21]. The brains were harvested two days after surgery and were dissected and incubated with 4% paraformaldehyde for 24 h, then dehydrated in 30% sucrose solution for 48 h until sinking to the bottom. Twenty micrometer-thick coronal hippocampal cryosections were cut via cryostat (Leica, Wetzlar, Germany). Then sections were incubated with Iba-1 primary antibodies (1:100 dilution, Abcam, Cambridge, UK) overnight at 4 °C, followed by incubation with Alexa-Fluor 488 secondary antibodies (1:500 dilution, Abcam, Cambridge, UK) for 60 min. Nuclei were then counterstained with DAPI (1:5000 dilution, Roche, Mannheim, Germany). Images were captured by TCS SP8 X confocal fluorescence microscope (Leica, Wetzlar, Germany). Image J was used for the quantification of the positive percentage.

### Transmission electron microscopy

The brains were harvested two days after surgery. As described previously [9], sections of the hippocampal CA1 region were prepared for electron microscopy by fixation in 0.5% glutaraldehyde and 1% osmium tetroxide. Samples were dehydrated in acetone solutions at increasing concentrations, embedded in an epoxy resin, and stained with uranyl acetate and lead citrate. The ultrastructural changes of the basal lamina, tight junction and mitochondria, as well as the angioedema surrounding the capillaries all indicative of BBB integrity disruption were observed with JEM-1400 transmission electron microscopy (JEOL, Tokyo, Japan).

### Statistical analysis

Prism 7.0 software (GraphPad software, La Jolla, CA, USA) was used to analyze the data. Data were presented as means ± SEM. The power calculation was performed using information collected from a preliminary study. Based on the preliminary data, assuming a two-sided Student’s t-test, samples of six for biochemistry studies, and ten for Morris water maze test would lead to 90% power and 95% significance. Two-way repeated-measures ANOVA followed by post-hoc Bonferroni test was used to analyze the water maze escape latency. Other data were analyzed with two-way ANOVA with Post hoc Bonferroni test when data was normally distributed or Kruskal-Wallis test when data was not normally distributed. The normal distribution of data was tested with Kolmogorov-Smirnov test (p>0.1), and values of p<0.05 were considered statistically significant.

**Figure 1.**
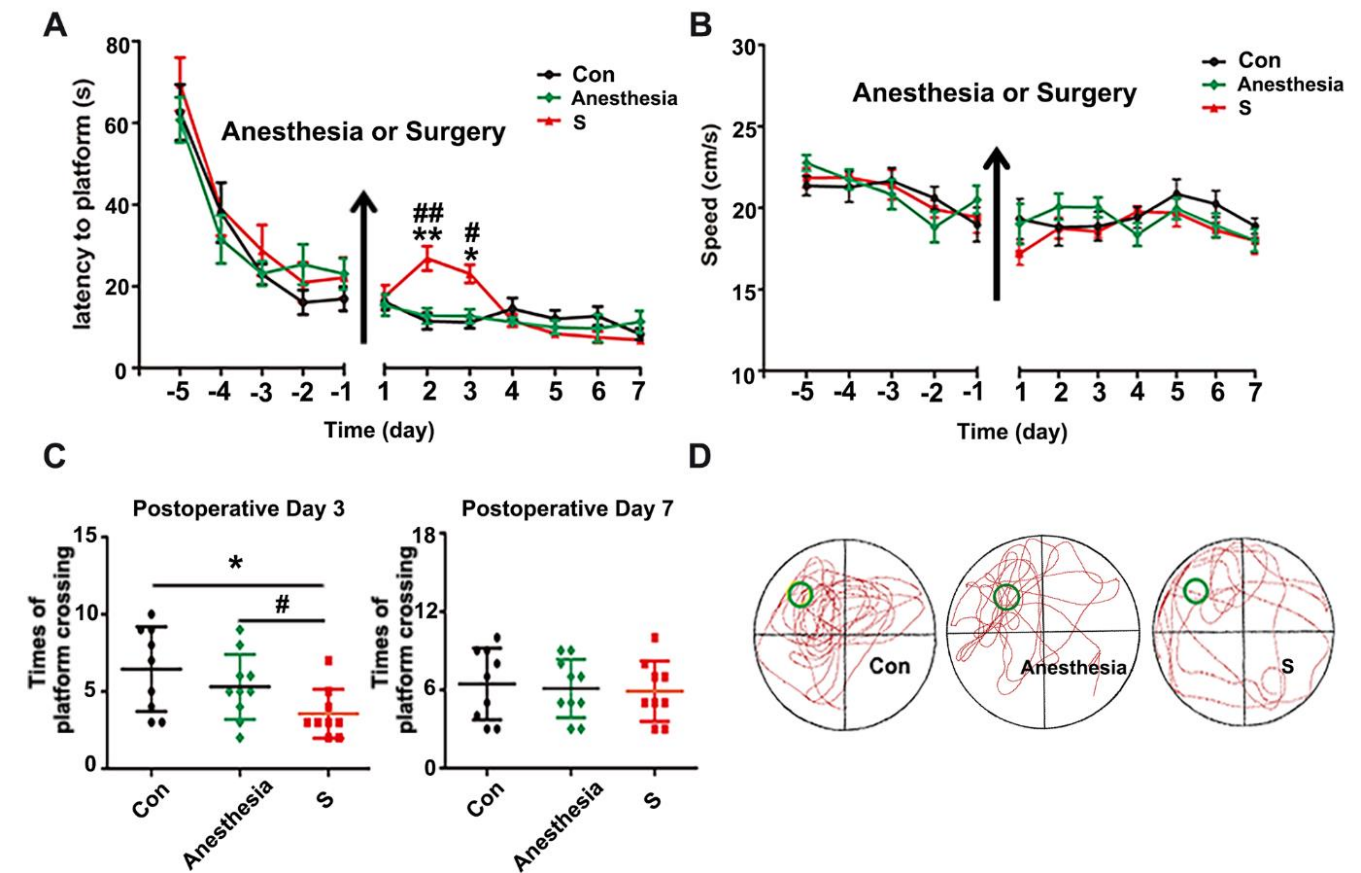
Surgery plus sevoflurane anesthesia, but not anesthesia alone, impaired spatial memory in APP/PS1 mice. (A) The mice in surgery (S) group, but not anesthesia group, had a longer escape latency on postoperative day two and three compared with control (Con) group. (B) There was no difference among three groups in swimming speeds during the training and probe tests. (C) The platform crossings of S group were significantly decreased on postoperative day three compared with Con group. (D) The trajectory of probe trial test of three groups on postoperative day three. Results were presented as mean ± SEM (n = 10). Two-way ANOVA with post-hoc Bonferroni test, *p<0.05, **p<0.01 compared with Con group, ^#^p<0.05,^# #^p<0.05 compared with anesthesia group.

## RESULTS

### Surgery plus anesthesia, but not anesthesia alone, impaired spatial memory in APP/PS1 mice

To identify whether anesthesia alone or surgery plus anesthesia could induce cognitive dysfunction in APP/PS1 mice, Morris water maze test was used to evaluate memory function. The mice were randomly divided into three groups: Con (Control), anesthesia (30 min sevoflurane anesthesia) and S (partial hepatectomy plus anesthesia, without liver function alteration) group. There was no significant difference among the three groups in water maze training before intervention (p>0.05). However, the mice in S group showed a significant increase in the escape latency time on the 2^nd^ and 3^rd^ day after surgery compared with Con group (p<0.05, Fig. 1A). The platform crossings of S group significantly decreased on the 3^rd^ day after surgery compared with Con group (Fig. 1C and D, p<0.05). Sevoflurane anesthesia alone did not significantly affect water maze test results compared with Con group (p>0.05, Fig. 1A and C). There was no significantly difference among the three groups in swimming speed during the training and probe tests (p>0.05, Fig. 1B), which excluded the effects of motor and perceptual abilities on spatial memory after intervention. These results suggested that surgery, but not anesthesia, impaired spatial memory in APP/PS1 mice.

**Figure 2.**
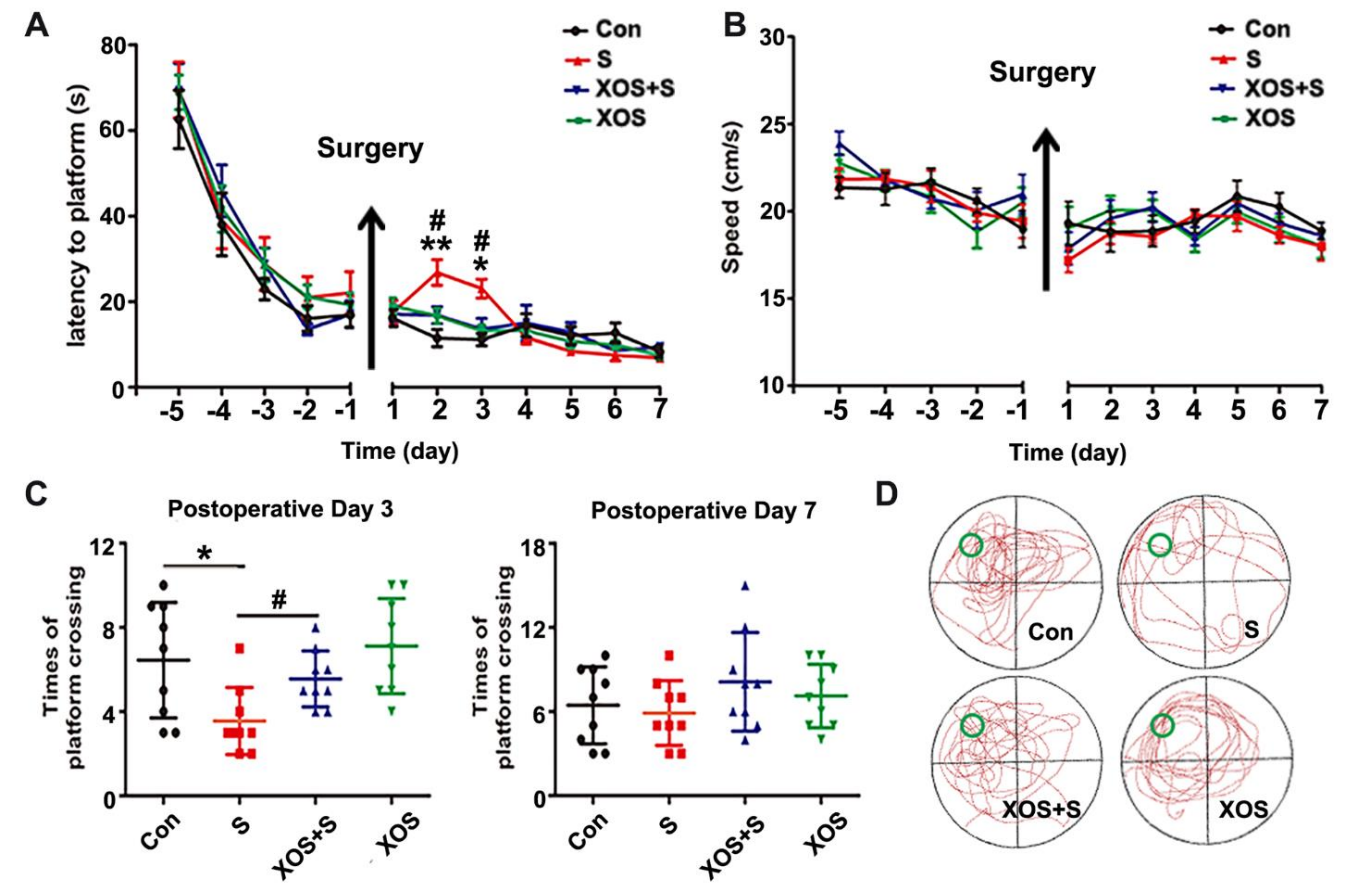
XOS intervention attenuated spatial memory deficit of APP/PS1 mice induced by surgery. (A) The mice in S group had a longer escape latency on postoperative day two and three than Con group. (B) There was no difference among four groups in swimming speeds during the training and probe tests. (C) The platform crossings in S group were decreased on postoperative day three compared with Con or XOS+S groups. (D) The trajectory of probe trial test of 4 groups on postoperative day three. Results were presented as mean ± SEM (n = 10). Two-way ANOVA with post-hoc Bonferroni test, *p<0.05, **p<0.01 compared with Con group, ^#^p<0.05 compared with S group.

### XOS intervention attenuated spatial memory deficit of APP/PS1 mice induced by surgery

Prebiotics could inhibit the reproduction of pathogenic bacteria and have shown beneficial effects to the cognitive function [3, 15]. In the present study, the mice were randomly divided into four groups: Con, S, XOS+S and XOS groups, and the mice in XOS and XOS+S group were given XOS by gavage once a day through the entire course of six weeks. One week before the surgery, we performed the water maze test. There was no significant difference among Con, S, XOS+S, and XOS groups in water maze training test before surgery (p>0.05), which indicated that XOS alone did not influence cognitive function. However, mice in S group showed a significant increase in the escape latencies on the 2^nd^ and 3^rd^ day after surgery compared with Con group (p<0.05, Fig. 2A). The platform crossings in S group significantly decreased on the 3^rd^ day after surgery compared with Con group (p<0.05, Fig. 2C). XOS intervention reversed the decreased platform crossings on the 3^rd^ day after surgery and the increase of escape latencies on postoperative day two and three (p<0.05). However, there was no significant difference in escape latencies and platform crossings between Con and XOS group (p>0.05, Fig. 2A and C). The swimming speed during the training and escape latency tests were comparable for the four groups (p>0.05, Fig. 2B). These data indicate that XOS can attenuate spatial memory dysfunction induced by surgery.

**Figure 3.**
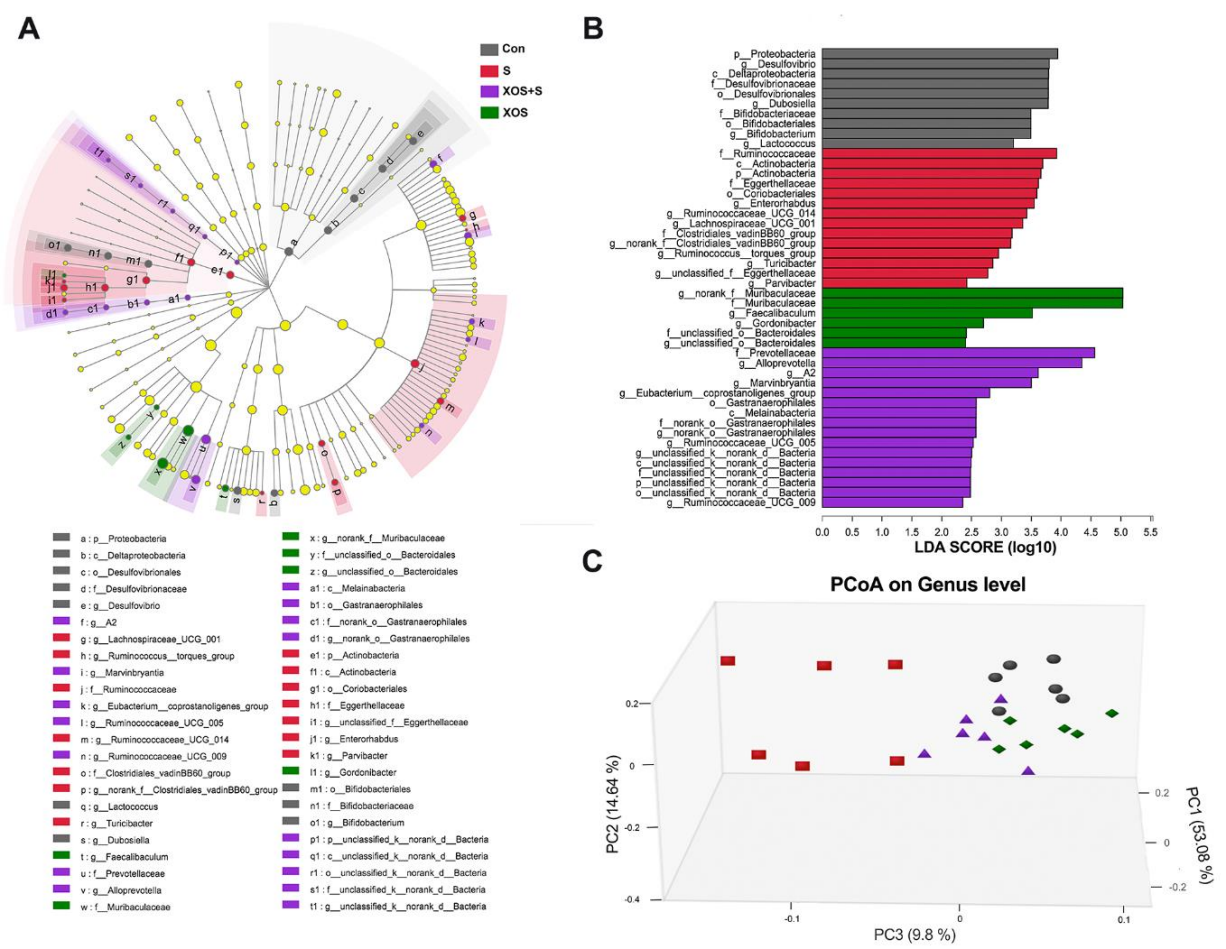
XOS intervention attenuated intestinal microbiota alteration induced by surgery. (A) Taxonomic cladogram obtained from linear discriminant analysis effect size (LEfSe). Biomarker taxa are highlighted by colored circles and shaded areas. The diameter of each circle reflects the abundance of that taxa in the community. (B) Taxa with a different abundance among four groups, and a total of 46 taxa were screened out with a linear discriminant analysis threshold score of 2.0. (C) The principal co-ordinates analysis (three-dimensional) of the intestinal microbiome composition on the genus level based on the weighted-unifrac distance among the four groups (n=6), Kruskal-Wallis test p<0.05 and log 10 LDA threshold=2.

### XOS intervention attenuated intestinal microbiota alteration induced by surgery

In order to study whether surgery could affect intestinal microbiota and the effects of XOS during the process, we performed 16S rRNA gene sequencing in fecal samples collected from Con, S, XOS+S and XOS groups on the 2^nd^ and 3^rd^ day after surgery when spatial memory dysfunction occurred. The alterations of the intestinal microbiota among four groups from phylum to genus were analyzed using linear discriminant analysis effect size (LEfSe) [22]. In figure 3A and B, a total of 46 taxa were screened out with a linear discriminant analysis as threshold score is 2. Taxa higher abundance in the Con group mainly belonged to phylum *Proteobacteria* and *Actinobacteria*, class *Deltaproteobacteria* and *Actino-bacteria*, order *Bifidobacteriales*, *Desulfo-vibrionales* and *Coriobacteriales*, genus such as *Desulfovibrio*, *Dubosiella* and *Bifidobacterium*. Taxa higher abundance in the S group mainly belonged to phylum *Actinobacteria*, class *Actinobacteria*, order *Coriobacteriales*, family *Ruminococcaceae* and *Eggerthellaceae*, genus such as *Actinobacteria*, and *Ruminococcaceae_UCG_014*. Taxa higher abundance in the XOS+S group mainly belonged to class *Melainabacteria*, order *Gastranaerophilales*, family *Prevotellaceae*, genus *Alloprevotell*a, *A2* and *Ruminococcaceae_UCG_009*. Taxa higher abundance in the XOS group mainly belonged to family *Muribaculaceae* and *unclassified Bacteroidales*, genus *Faecalibaculum* and *Gordonibacter*.

**Figure 4.**
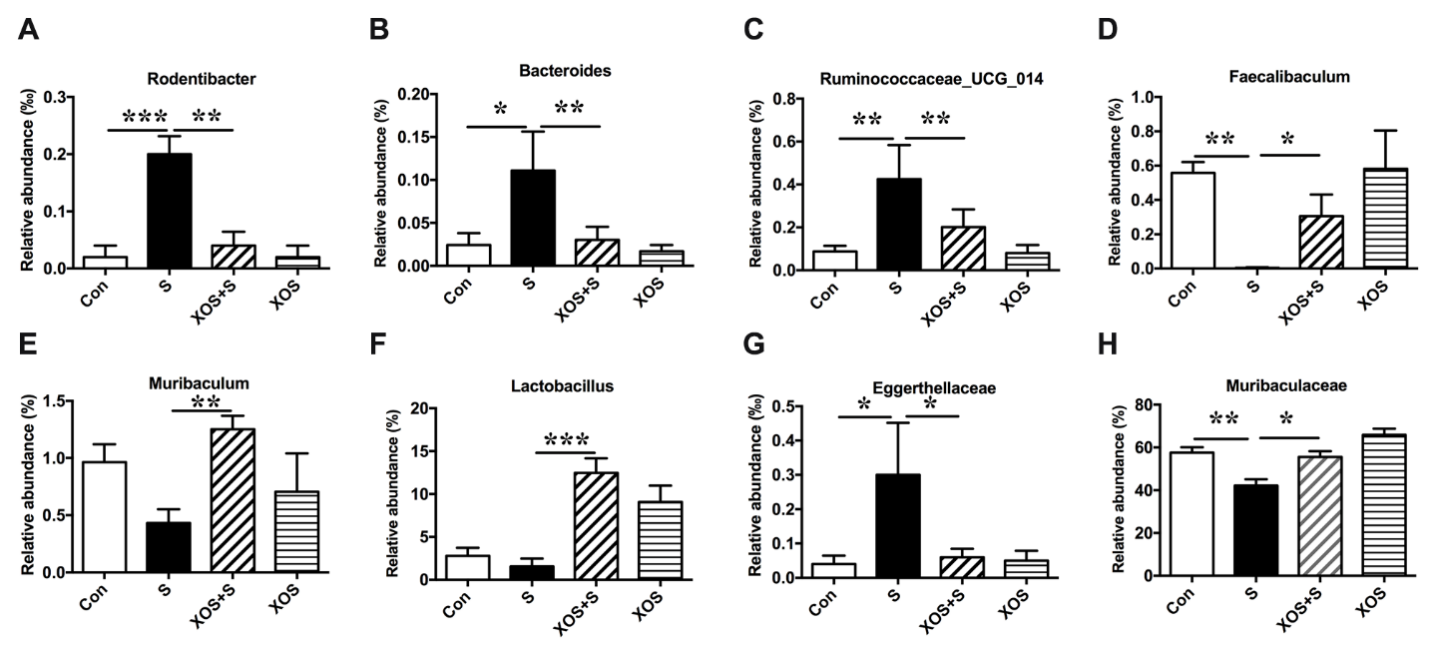
XOS intervention attenuated intestinal microbiota alteration induced by surgery Chart of the relative abundance of the differential levels of bacteria at the genus and family level. (A-C) Three genera (*Rodentibacter, Bacteroides, Ruminococcaceae_UCG_014*) were increased in S group compared with Con group, and XOS intervention attenuated the increase of these three genera after surgery. (D) One genus (*Faecalibaculum)* was decreased in S group compared with Con group, and XOS intervention attenuated the decrease of this genus after surgery. (E and F) Two genus (*Muribaculum* and *Lactobacillus*) were increased in XOS+S group compared with S group. (G) One family (*Eggerthellaceae*) was increased in S group compared with Con group, and XOS intervention attenuated the decrease of this genus after surgery. (H) One family (*Muribaculaceae*) was decreased in S group compared with Con group, and XOS intervention attenuated the decrease of this genus after surgery. Results were presented as mean ± SEM (n = 6). Two-way ANOVA with post hoc Bonferroni test, *p<0.05; **p<0.01; ***p<0.001.

Next, we analyzed the β diversity of the four groups. The principal co-ordinates analysis (PCoA) showed that the composition of intestinal bacteria in S group was distinguished from Con group, and XOS+S group was distinguished from S group, but there was no obvious difference between XOS and Con groups (Fig. 3C). Furthermore, we found that eight taxons changed significantly among four groups. Three genera (*Rodentibacter, Bacteroides, Ruminococcaceae_UCG_014*) were significantly increased in S group compared with Con group (p<0.05, Fig. 4A-C). XOS intervention attenuated the increased genera in S group (p<0.05, Fig. 4A-C), but not in control condition (p>0.05, Fig. 4A-C). One genus (*Faecalibaculum*) was significantly decreased in S group compared with Con group (p<0.05, Fig. 4D). XOS intervention significantly attenuated the decrease of this genus in S group (p<0.05, Fig. 4D), but not in control condition (p>0.05, Fig. 4D). Two genera (*Muribaculum* and *Lactobacillus*) were significantly increased in XOS+S group compared with S group (p<0.05, Fig. 4E and F), but there was no significant difference between S and Con groups, or between XOS and Con groups (p>0.05, Fig. 4E and F). One family (*Eggerthellaceae*) was significantly increased in S group compared with Con group (p<0.05, Fig. 4G), and XOS intervention significantly attenuated the increase of this family after surgery (p<0.05, Fig. 4G). One family (*Muribaculaceae*) was significantly decreased in S group compared with Con group (p<0.05, Fig. 4H), and XOS intervention significantly attenuated the decrease of this family after surgery (p<0.05, Fig. 4H).

Collectively, these data indicate that surgery altered the composition and abundance of intestinal microbiota, and XOS could modulate surgery induced microbiota alternations. Furthermore, the intestinal microbiota alterations might be involved in surgery induced cognitive dysfunction.

### XOS intervention attenuated intestinal inflammation and barrier integrity damage induced by surgery

**Figure 5.**
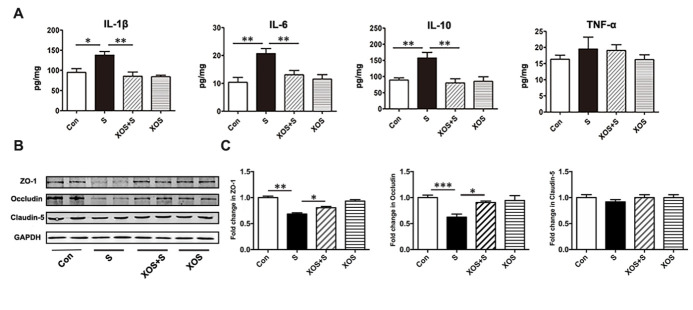
XOS intervention attenuated intestinal inflammation and barrier integrity damage induced by surgery. (A) IL-1β, IL-6 and IL-10 levels were increased in the intestine of mice in S group compared with Con group, and XOS intervention ameliorated the increase of IL-1β, IL-6 and IL-10 after surgery. (B and C) Western blot analysis showed that tight junction protein levels of ZO-1 and Occludin were downregulated in S group compared with Con group, and XOS increased the expression of ZO-1 and Occludin after surgery. There was no difference among four groups for Claudin-5 expression. Results were presented as mean ± SEM (n = 6). Two-way ANOVA with post hoc Bonferroni test, *p<0.05, **p<0.01.

To investigate whether surgery stimulation could induce intestinal inflammatory responses and whether XOS intervention could be protective, several classical inflammatory mediators were tested. As shown in Fig. 5A, pro-inflammatory mediator IL-1β, IL-6, and immunosuppressive mediator IL-10 significantly increased in the colon of mice in S group compared with Con group (p<0.05). XOS intervention ameliorated the increase of IL-1β, IL-6, and IL-10 expressions after surgery (p<0.05), but not in control condition (p>0.05). Nevertheless, pro-inflammatory mediator TNF-α did not change among the 4 groups (p>0.05).

To investigate the effects of XOS intervention on surgery stimulation induced intestinal barrier integrity variation, several critical proteins important for maintaining intestinal barrier integrity were tested. Western blotting analysis showed that the expressions of tight junction protein ZO-1 and Occludin significantly decreased in the S group compared with Con group (p<0.05), while XOS intervention increased the expression of ZO-1 and Occludin after surgery (p<0.05), but not in control condition (p>0.05, Fig. 5B and C). However, there was no significant difference among four groups for Claudin-5 (p>0.05, Fig. 5B and C). These results suggested that surgery could induce intestinal inflammatory response and intestinal barrier integrity disruption. XOS modulated intestinal microbiota and alleviated the intestinal barrier damage and inflammatory response induced by surgery.

### XOS intervention attenuated BBB disruption induced by surgery

**Figure 6.**
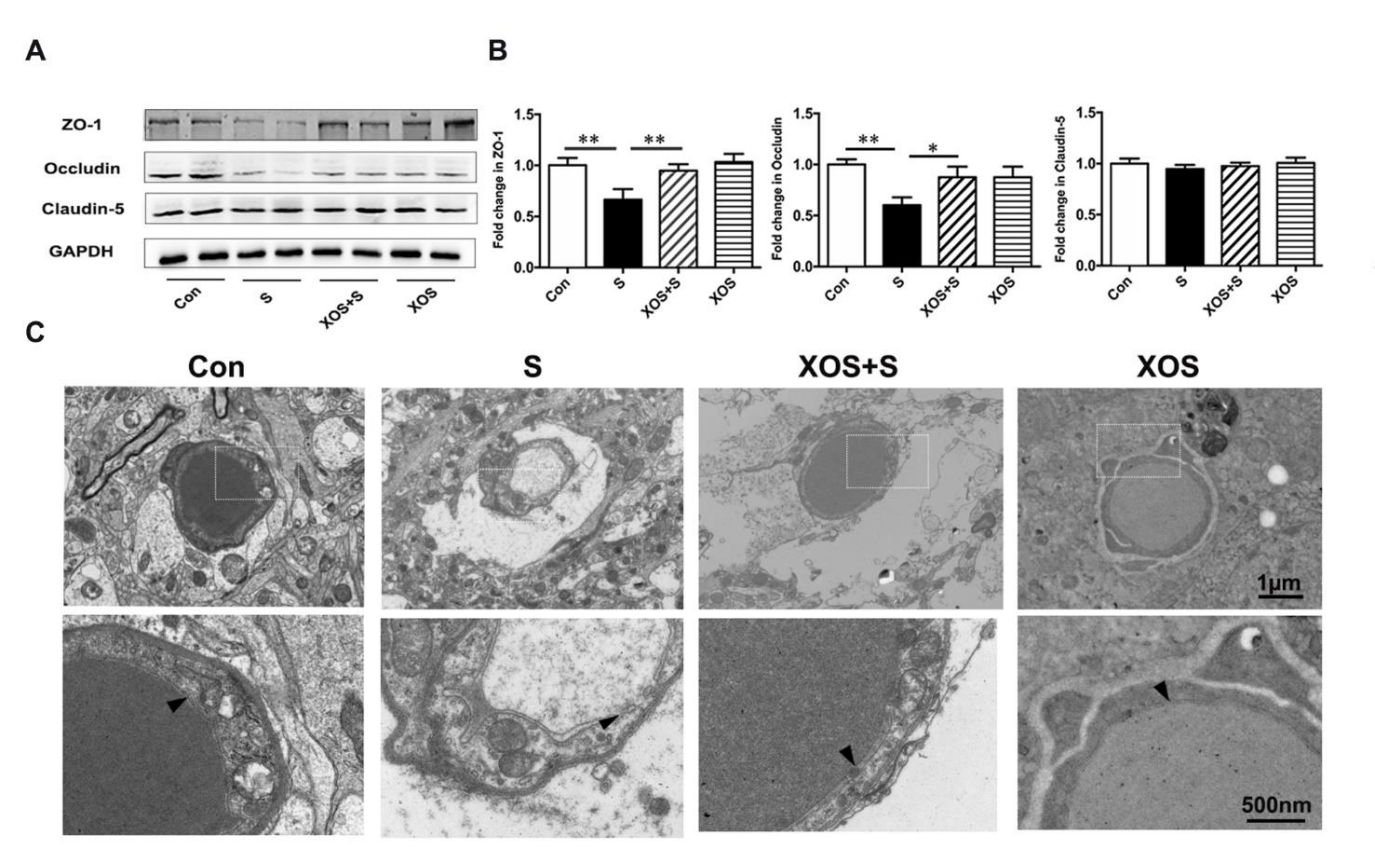
XOS intervention attenuated BBB disruption induced by surgery. (A and B) There was a decrease in the expression of tight junction proteins ZO-1 and Occludin in S group compared with Con group, and XOS intervention increased the expression of ZO-1 and Occludin after surgery. There was no difference among four groups for Claudin-5 expression in the hippocampus. (C) The transmission electron microscopy showed that the ultrastructure of the basal laminas of mice in Con group was continuous and integrated, while the mice in S group had BBB impairments on postoperative day two. XOS intervention ameliorated the disruption of the BBB ultrastructure after surgery. The arrow heads represent the basal laminas of BBB. Results were presented as mean ± SEM (n = 6). Two-way ANOVA with post hoc Bonferroni test, *p<0.05, **p<0.01.

We further investigated whether surgery stimulation could induce BBB disruption in the hippocampus, and the effects of XOS intervention. The critical BBB components, tight junction proteins were analyzed. As shown in Fig. 6, there was a significant decrease in tight junction protein ZO-1 and Occludin expression in S group compared with Con group (p<0.05). XOS intervention increased the expression of ZO-1 and Occludin after surgery (p<0.05), but not in control condition (p>0.05). In addition, there was no significant difference of Claudin-5 expression among 4 groups in the hippocampus (p>0.05, Fig. 6B and C).

We also performed the morphological analysis of the BBB with transmission electron microscopy. The results showed that the ultrastructure of the basal laminae of mice in Con group was continuous and integrated, the tight junctions and endothelial cells were normal. The mice in S group showed BBB ultrastructure impairments on the 2^nd^ day after surgery, which manifested as collapsed local basal laminae and opened tight junctions. XOS intervention ameliorated BBB impairments after surgery, including both the collapsed basal laminae and opened tight junctions. Moreover, there was no obvious ultrastructure difference between XOS and Con groups (Fig. 6C).

### XOS intervention attenuated neuroinflammatory response induced by surgery

**Figure 7.**
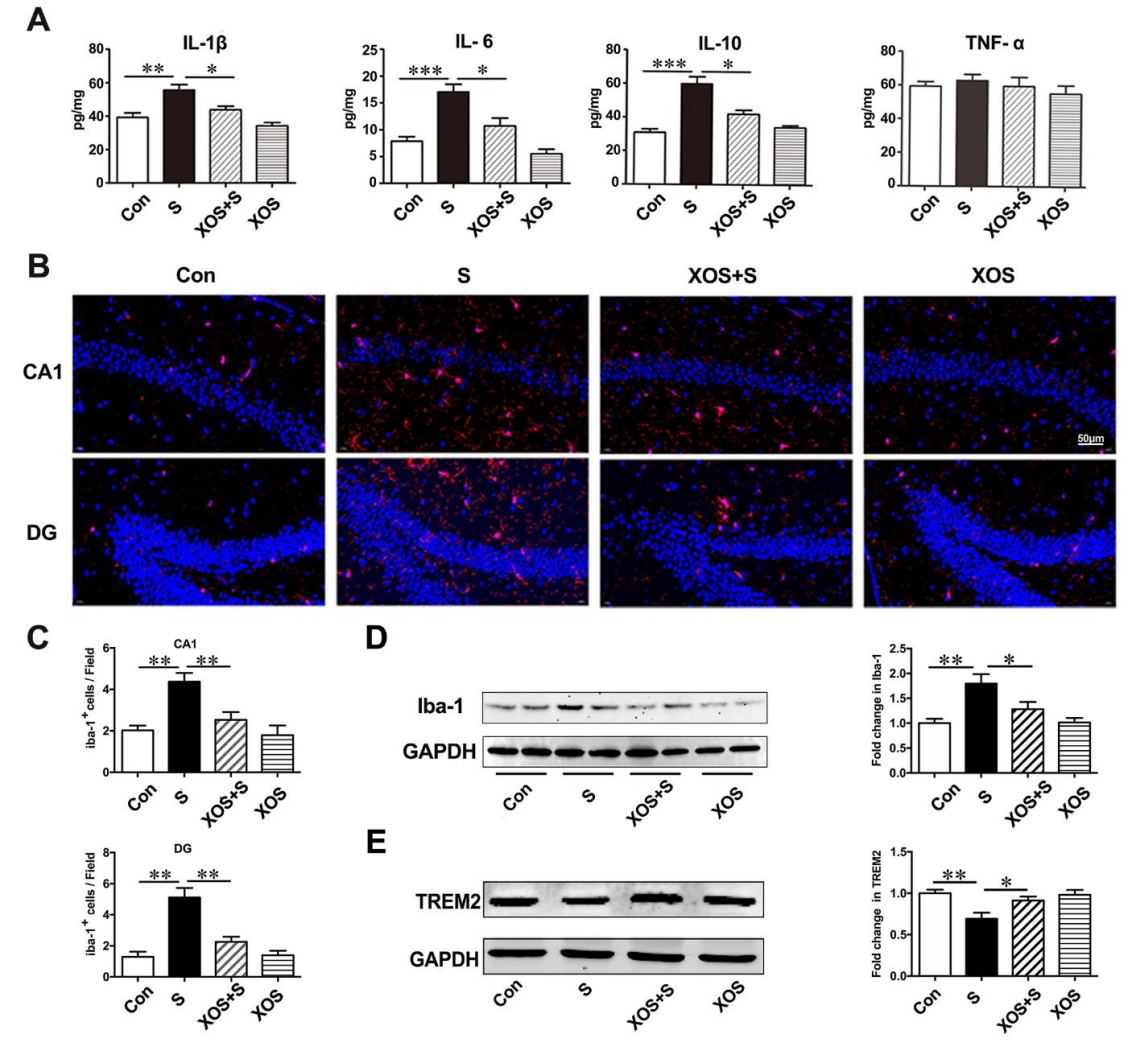
XOS intervention attenuated neuroinflammatory response induced by surgery. (A) IL-1β, IL-6 and IL-10 levels were increased in the hippocampus of mice in the S group compared with Con group, and XOS intervention attenuated the increase of IL-1β, IL-6 and IL-10 after surgery. The level of TNF-α was not changed among four groups. (B and C) The immunofluorescence showed an increase of Iba-1 positive cells in CA1 region and dentate gyrus in S group compared with Con group, and XOS intervention attenuated the increase of Iba-1 positive cells after surgery. (D) Western blot analysis showed that Iba-1 expression was increased in S group compared with Con group, and XOS intervention attenuated the increase of Iba-1 expression after surgery. (E) The expression of TREM2 was decreased in S group compared with Con group, and XOS intervention attenuated the decrease of TREM2 expression after surgery. Results were presented as mean ± SEM (n = 6). Two-way ANOVA with post hoc Bonferroni test, *p<0.05, **p<0.01, ***p<0.001.

With the BBB disruption in the hippocampus, we further investigated whether surgery stimulation could induce neuroinflammatory response in the hippocampus, as well as the effects of XOS intervention during the process. The results showed that the levels of IL-1β, IL-6, and IL-10 significantly increased in the hippocampus of mice in S group compared with Con group (p<0.05). XOS intervention attenuated the increased levels of IL-1β, IL-6, and IL-10 after surgery (p<0.05), but not in control condition (p>0.05). The level of TNF-α did not change among the four groups (p>0.05, Fig. 7A).

Iba-1 is a specific protein expressed by microglia [23], and used as the indicator of microglia in the present study. The immunofluorescence staining showed a significant increase of Iba-1 positive cells in CA1 subfield and DG in hippocampus in S group compared with Con group (p<0.05). XOS intervention attenuated the increased Iba-1 positive cells after surgery (p<0.05), but not in control condition (p>0.05, Fig. 7B and C). Then, we quantitated Iba-1 expression with western blotting analysis. The results showed that the expression of Iba-1 was significantly increased in S group compared with Con group (p<0.05). XOS intervention attenuated the increase of Iba-1 expression after surgery (p<0.05), but not in control condition (p>0.05, Fig. 7D).

TREM2 is a class of immunoglobulin-like receptors highly expressed in microglia, which can regulate the microglia polarization and modulate the inflammatory response in CNS [24, 25]. We found the expression of TREM2 significantly decreased in S group compared with Con group (p<0.05). XOS intervention attenuated the decrease of TREM2 expression after surgery (p<0.05), but not in control condition (p>0.05, Fig. 7E). These results indicate that surgery increased IL-1β, IL-6 and IL-10 expressions, decreased TREM2 expression and microglia activation, and TREM2 expression changes could be the upstream mechanisms for microglia activation during the process. Furthermore, XOS intervention could attenuate these processes, which suggests that the neuroinflammatory response was dependent on intestinal microbiota alterations.

## DISCUSSION

**Figure 8.**
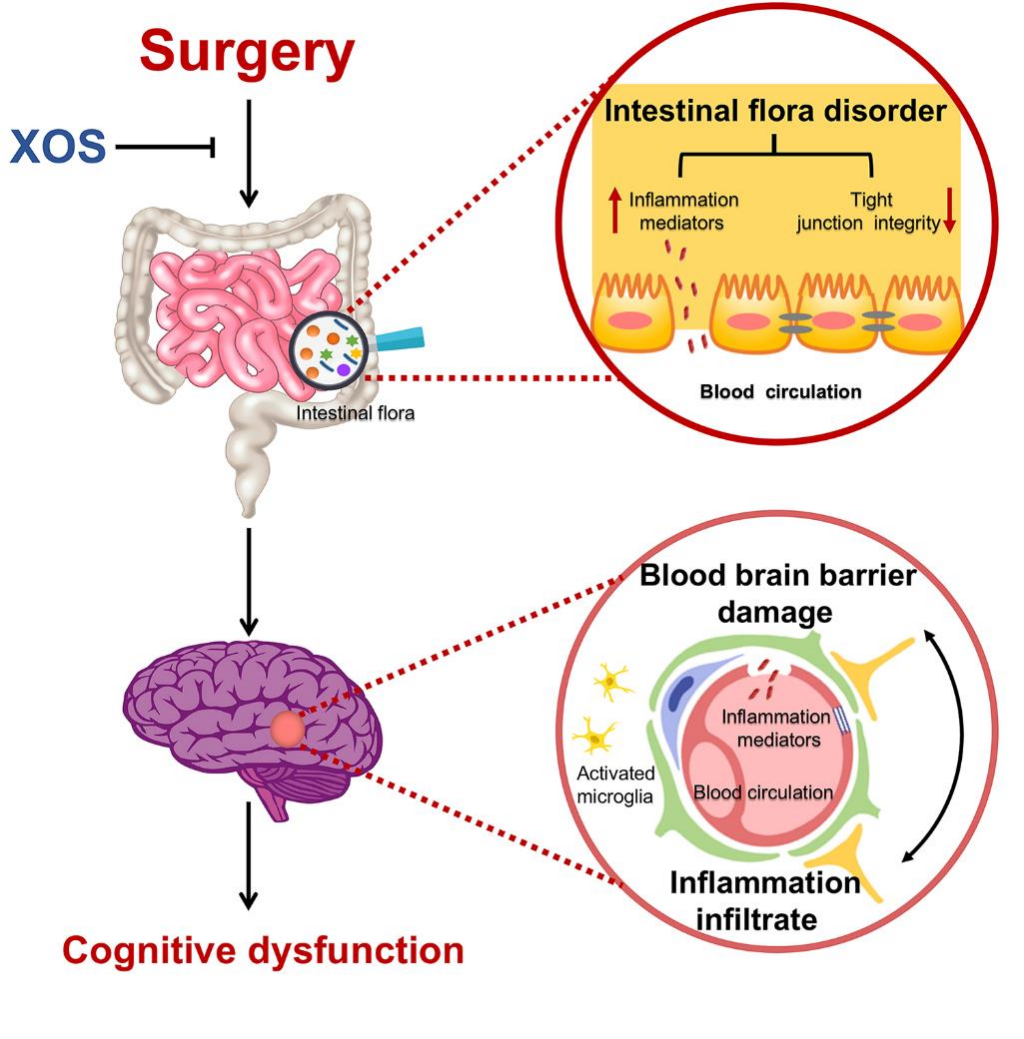
Schematic illustration of the proposed intestinal microbiota related mechanisms underlying cognitive dysfunction in APP/PS1 mice after surgery stimulation (partial hepatectomy). Surgery stimulation (partial hepatectomy) induces intestinal dysbiosis, intestinal inflammatory response and barrier damage, then hippocampal blood brain barrier damage and neuroinflammatory response, and results in cognitive dysfunction in in APP/PS1 mice. XOS stabilizes intestinal microbiota and inhibits the pathophysiological process after surgery and protects the cognitive function.

The present results indicate that surgery stimulation with anesthesia, but not anesthesia alone, induced spatial memory impairment in APP/PS1 mice. Furthermore, surgery altered intestinal microbiota, damaged intestinal barrier and BBB integrity, and triggered peripheral and central inflammatory responses. Prebiotics intervention could stabilize intestinal microbiota, improve intestinal barrier and BBB integrity, and prevent peripheral inflammatory mediators from infiltrating into the hippocampus. Thus, prebiotics intervention improved spatial memory impairment induced by surgery (Fig. 8).

The PCoA in β diversity analysis showed that the intestinal microbiota distribution after surgery was distinguished from normal condition, which indicates that surgery stimulation disrupted the intestinal microbiota balance in APP/PS1 mice. The typical upregulated microbiota after surgery include *Rumino-coccaceae_UCG_014*, *Rodentibacter*, *Eggerthe-llaceae*, and *Bacteroide*, and downregulated microbiota include *Faecalibaculum* and *Muribaculaceae*. XOS invention could significantly increase the richness of *Muribaculum* and *Lactobacillus* after surgery. Previous studies indicate that *Ruminococcaceae* in intestine is related to certain gastrointestinal diseases and metabolic diseases [26, 27]. *Ruminococcaceae* is increased in AD mice, suggesting that *Ruminococcaceae* may participate in the process of cognitive dysfunction the neurodegenerative disease [28]. *Rodentibacter* is present in both mouse and rat, and the rpoB gene sequence of this strain shared 86?% or higher similarity with *Pasteurellaceae*, indicating that there is a genus-level relationship in *Pasteurellaceae* [29]. In previous study, the increase in *Pasteurellaceae* was positively correlated with inflammatory bowel disease [30]. *Faecalibaculum* is a gram-positive obligate anaerobe and has the ability of higher fermentation to increased short chain fatty acids especially butyrate production and carbohydrate metabolism [31, 32]. Reduced *Faecalibaculum* has been found in Parkinson’s disease [33]. The decrease of *Muribaculum* has been found in the studies of ileitis and T cell-induced colitis [34, 35]. Similar to the present results, there is no evidence for the change of *Lactobacillus* in AD and Parkinson’s disease, although it is beneficial in neurodegenerative diseases [36-38] and could reduce intestinal dysfunction in gluten-specific related immune responses [39]. XOS is not food for *lactobacillus*, but previous studies showed that XOS intervention could increase the level of *lactobacillus* in mice and pigs [13, 40]. The possible reason is that XOS could modulate the intestinal environment, while improved intestinal environment is beneficial for *lactobacillus*survival. In summary, the typical changed intestinal taxa after surgery also play roles in the neurodegeneration diseases and immune diseases, indicating that intestinal microbiota alterations are the potential mechanism for the intestinal barrier damage and inflammatory response, as well as cognitive dysfunction after surgery.

When compared with other well stablished prebiotics, such as inulin or FOS, XOS showed higher resistance to digestion and has been reported to present many beneficial functions including preventing intestinal microbiota alteration, neurotoxicity and intestinal inflammation [41, 42]. The present study indicates that XOS intervention attenuated surgery induced intestinal microbiota alterations, including four upregulated and two downregulated taxa, then *Muribaculum* and *Lactobacillus*were also upregulated by XOS intervention after surgery.

Previous studies have shown that numerous diseases are related with intestinal dysbiosis, such as gastrointestinal diseases, metabolic diseases and neurodegenerative diseases [43-45]. Intestinal dysbiosis is related to intestinal inflammation and barrier integrity [46, 47], and may affect CNS via gut-brain axis [48, 49]. The gastrointestinal system is the largest immune organ in the human body, meanwhile, the intestinal immune system as well as intestinal and blood brain barriers are two critical communication routes between intestinal microbiota and brain [50, 51]. The present results showed that tight junction protein ZO-1 and Occludin of intestinal epithelial cells were downregulated, and the inflammation mediator IL-1β, IL-6 and IL-10 were upregulated after surgery. TNF-α level peaked on the 1^st^ day after surgery as indicated by our previous study [52]. In the present study, the levels of inflammatory cytokines were tested on the 2^nd^ day after surgery when cognitive dysfunction occurred. Although TNF-α was slightly upregulated after surgery, there was no significant difference among four groups, which indicated that TNF-α may not be the major inflammatory cytokines for the postoperative neuroinflammatory response and cognitive dysfunction in the present model. Furthermore, intestinal barrier damage and increased inflammation response can be attenuated by XOS intervention through modulating the intestinal microbiota, which is consistent with the anti-autoimmune effect of XOS in non-obesity-diabetes mice [53]. Evidence has proven that XOS intervention can reduce intestinal permeability markers and inflammatory response through inhibiting of natural killer T cells and cytotoxic T cells [53], which require further investigations in the perioperative context.

Pathogens and inflammation mediators could cross the ‘leaky gut’ into blood circulation and be the underlying mechanism for the interaction between intestinal microbiota alteration and CNS dysfunction [54, 55]. The intestinal barrier and BBB play a predominantly protective role in the communication between the periphery and CNS [56, 57]. Once the protective shields are damaged, the pathogen and inflammatory mediators could infiltrate into the CNS through peripheral circulation, then activate microglia and affect cognitive function [58]. The present study showed that surgery induced tight junction protein ZO-1 and Occludin downregulation, BBB integrity damage, and triggered neuroinflammatory response in the hippocampus. These pathological processes could be related to intestinal dysbiosis and ‘leaky gut’ after surgery.

The present study showed the number of Iba-1 positive cells increased, and the soma of microglia enlarged in the hippocampus after surgery. Consistent with previous study results, these changes indicate microglia activation [59, 60]. The expression of TREM2 was decreased after surgery. TREM2 is a transmembrane receptor protein and expresses specifically by the microglia [61]. It could relieve neuroinflammatory response and improve cognitive function in both neurodegenerative diseases and brain injury model [62, 63]. The deficiency of TREM2 could affect the microglia function, aggravate the β amyloid and cause α-synuclein overexpression in neurodegenerative disease [63, 64]. XOS intervention ameliorated the trends of microglia activation and TREM2 expression, indicating that TREM2-microglia signaling were related to intestinal dysbiosis after surgery.

Growing evidences support that intestinal microbiota is extremely important for maintaining CNS function and intestinal dysbiosis could affect cognitive function during multiple chronic CNS diseases [65, 66]. Previous studies indicated that intestinal microbiota could regulate CNS through neural, metabolic, endocrine and immunological mechanisms [67]. The enteric nervous system could convey intestinal metabolite and sensory information to the CNS through the receptors expressed on vagal afferent nerves. Recently, an expert insight indicated that intestinal microbiota-bile acid-brain axis may participate in the development and progression of AD [68]. Also, the intestinal microbiota plays a role in the regulation of the hypothalamic-pituitary-adrenal axis and is critical for the stress response [69]. Finally, evidences have proved that intestinal microbiota can modulate neuroinflammatory response, and the fact that diseases or the complications with cognitive dysfunction such as AD, PD, depression, as well as postoperative dysfunction in the present study are closely correlated with neuroinflammatory response [65, 70, 71]. The previous study showed that the plasma inflammatory cytokine changed significantly in the first 24 h, but not obvious afterwards. In the central nerve system, the inflammatory response could persist longer, on the 2^nd^ day after surgery in our study and even longer in the previous study [72]. The model used in the present study was six-month-old male APP/PS1 mice with fragile brain, in which the neuroinflammatory response would persist longer [25]. Our previous study also indicated that BBB disruption during perioperative would persist for more than two days [73], which may also participate the neuroinflammatory response in this study. Therefore, to understand the function of multiple pathways between fecal microbiota and cognitive function could be the direction for future studies in the area.

Here we found that surgery stimulation induced intestinal microbiota alterations, reduced tight junction protein ZO-1 and Occludin expressions, damaged intestine barrier and BBB, increased peripheral and CNS inflammation response, and resulted in cognitive dysfunction. Furthermore, XOS intervention modified intestinal alteration, protected the intestinal barrier and BBB integrity, attenuated inflammatory response, and reversed POCD in APP/PS1 mice. Therefore, intestinal microbiota is also important for maintaining CNS function during perioperative context as gut-brain axis plays critical roles during the pathophysiological process of POCD, and prebiotics supplementation could be a valuable preventative approach.
